# Psychometric approaches to defining cognitive phenotypes in the Old Order Amish

**DOI:** 10.1002/gps.5903

**Published:** 2023-04

**Authors:** Andrew Zaman, Laura Caywood, Michael Prough, Jason Clouse, Sharlene Harrington, Larry Adams, Denise Fuzzell, Sarada Fuzzell, Renee Laux, Sherri D. Hochstetler, Paula Ogrocki, Alan Lerner, Jeffery M. Vance, Jonathan L. Haines, William K. Scott, Margaret A. Pericak-Vance, Michael L. Cuccaro

**Affiliations:** 1 John P. Hussman Institute for Human Genomics, University of Miami Miller School of Medicine, Miami, Florida, USA; 2 Department of Population and Quantitative Health Sciences, Case Western Reserve University, Cleveland, Ohio, USA; 3 Department of Neurology, Case Western Reserve University, Cleveland, Ohio, USA; 4 Dr. John T. Macdonald Foundation Department of Human Genetics, University of Miami Miller School of Medicine, Miami, Florida, USA; 5 Cleveland Institute for Computational Biology, Case Western Reserve University, Cleveland, Ohio, USA

**Keywords:** Alzheimer’s, Amish, cluster, cognition, phenotype, psychometric

## Abstract

**Objective:**

Memory and cognitive problems are central to the diagnosis of Alzheimer’s disease (AD). Psychometric approaches to defining phenotypes can aid in identify genetic variants associated with AD. However, these approaches have mostly been limited to affected individuals. Defining phenotypes of both affected and unaffected individuals may help identify genetic variants associated with both AD and healthy aging. This study compares psychometric methods for developing cognitive phenotypes that are more granular than clinical classifications.

**Methods:**

682 older Old Order Amish individuals were included in the analysis. Adjusted *Z*-scores of cognitive tests were used to create four models including (1) global threshold scores or (2) memory threshold scores, and (3) global clusters and (4) memory clusters. An ordinal regression examined the coherence of the models with clinical classifications (cognitively impaired [CI], mildly impaired [MI], cognitively unimpaired), *APOE-e4*, sex, and age. An ANOVA examined the best model phenotypes for differences in clinical classification, *APOE-e4*, domain *Z*-scores (memory, language, executive function, and processing speed), sex, and age.

**Results:**

The memory cluster identified four phenotypes and had the best fit (*χ*^*2*^ = 491.66). Individuals in the worse performing phenotypes were more likely to be classified as CI or MI and to have *APOE-e4*. Additionally, all four phenotypes performed significantly differently from one another on the domains of memory, language, and executive functioning.

**Conclusions:**

Memory cluster stratification identified the cognitive phenotypes that best aligned with clinical classifications, *APOE-e4,* and cognitive performance We predict these phenotypes will prove useful in searching for protective genetic variants.

## 1 | INTRODUCTION

Alzheimer disease (AD) and related dementias affect large segments of the older population in the United States.^1^ Several well-established risk factors increase the likelihood for developing AD including age, genetic variation, and cerebrovascular disease.^2^ Equally compelling work is ongoing to identify genetic and environmental factors that confer protection against AD.^3^ Genetic studies of AD rely heavily on clinical classifications (e.g., AD, mild cognitive impairment, affected vs. unaffected, etc.). Typically, clinical classifications are derived from multiple sources including history of memory problems and decline, physical findings and medical history, subjective and informant-reported memory concerns, and cognitive testing. While clinical classifications are useful phenotypes for genetic studies of AD, the use of cognitive phenotypes (derived from cognitive testing) have been useful in detecting associations of genes with different cognitive functions as risk or protective factors.^4,5^ For this study, we will apply psychometric approaches to define cognitive phenotypes that can be applied in our genetic studies of AD among the Old Order Amish (OOA).

The development of cognitive phenotypes is typically predicated on the research question. For instance, do distinct cognitive profiles (i.e. patterns) differentiate classifications such as vascular dementia versus AD.^6^ Another common use is to differentiate broad classifications into meaningful subgroups that may have different etiologies or genetic risk. For example, individuals with mild cognitive impairment (MCI) represent a wide-ranging group that includes those with different patterns of cognitive performance (e.g., memory impairments vs. non-memory impairments vs. mixed). Finally, among individuals classified as “controls,” who present as seemingly unaffected, cognitive performance may be used to detect subtle patterns of changes that could be prodromal to dementia. Thus, psychometric approaches that use cognitive performance are valuable complements to clinical classification.

Some psychometric approaches (e.g., threshold and cluster stratification) using neuropsychological test performance have shown great promise in identifying genetic variants associated with AD.^4,5,7–10^ For example, employing a cognitively defined threshold stratification using *Z*-scores on single and multiple domains Mukherjee and colleagues constructed AD subgroups that were associated with different genetic variants.^5^ Specifically, in the memory only impaired subgroup, several well-known AD risk genes, *SORL1*, *Cass4*, and *CR1*, were associated with memory impairment. However, in the language impaired subgroup two genes, *SORL1* and *ZCWPW1*, were associated with better performance on the language composite, while two genes, *PTK2b* and *CD2AP* were associated with language impairment.

In contrast to defining subgroups based on impairment, defining subgroups of cognitively intact individuals has been limited but holds potential to determine genetic variants associated with healthy cognitive aging. There is a lot of variability in how normal or control phenotypes are defined, but most studies define the phenotype based on intact cognition and functional abilities.^11,12^ Multiple proposed protective factors for successful aging include cognitive and brain reserve,^13–15^ lifestyle factors,^16,17^ and lack of genetic risk variants such as those associated with AD in *APOE*, *ABCA7*, and *TREM2*.^18–21^ Focusing on cognition, it appears that among cognitively unimpaired (CU) individuals, factor analyses suggest that most of the variance in cognitive performance is explained by a single global factor.^22^ Thus, we hypothesize clustering by global cognitive performance is an effective method of developing refined cognitive phenotypes in both affected and unaffected individuals.

To test this hypothesis we will apply and compare psychometric methods (cluster vs. threshold-based stratification, and domain specific [e.g., memory] versus global based stratification) to identify the best approach to constructing cognitive phenotypes As a proof of concept, we will use our OOA dataset to compare these cognitive phenotypes to clinical classifications (cognitively unimpaired [CU], mildly impaired in cognition only [MI], and cognitively impaired [CI]), *APOE-e4* presence, and sociodemographic variables. We believe that clustering by global cognitive performance will refine cognitive phenotypes that align with and improve upon clinical classifications. Subsequent studies will test the association of these cognitive phenotypes with genetic variants in our OOA cohort.

## 2 | METHODS

### 2.1 | Participants

713 OOA community members were ascertained from Holmes County, Ohio and Adams, Elkhart, and LaGrange counties in Indiana for ongoing genetic studies of preserved cognitive ability. Participants have been ascertained for dementia, successful aging, and protective genetic factors. The OOA, an isolated founder population originating from the emigration of German and Swiss Anabaptists to the U.S. in the 1700’s and 1800’s,^23^ with fewer than 1000 founders.^24^ With self-imposed cultural and religious isolation, the introduction of genetic variation among the OOA has been significantly restricted. Anecdotally, their agrarian lifestyle and firm behavioral norms have reduced variation in environmental factors.

All participants were ascertained using information from Amish communities, published public directories, and referrals from previously enrolled participants^25–28^ Participants were included if they were (a) part of the OOA community and of OOA descent, (b) 75 years of age or older, (c) willing to participate in the study, (d) had relatives with AD, and (e) were not known to have dementia. All participants provided written informed consent and all procedures were approved by the Institutional Review Boards at the University of Miami Miller School of Medicine and Case Western Reserve University. All procedures performed were in accordance with the ethical standards of the institutions and with the Helsinki Declaration of 1975.

### 2.2 | Procedures

As part of their enrollment participants underwent a standard assessment that consisted of direct and informant report measures that assessed cognition (see next section: Cognitive Tests), living and functional skills (for example, AD8 checklist,^29^ Basic and Individual Activities of Daily Living^30^), and a demographic and medical history information (e.g. self-reported chronic illnesses). The informant measures were gathered from family members (e.g. spouses, siblings, and children) who have extensive knowledge of the participant. In addition to assessments and interviews, all newly enrolled participants were sampled to generate genetic data.

Clinical classifications were done by a panel consisting of neuropsychologists and neurologists with extensive experience in cognitive disorders of aging. The panel reviewed all clinical data and, using age, education, and gender adjusted cognitive norms, as well as consideration of sensory or motor problems, functional disabilities, and subjective memory complaints. Participants that displayed no cognitive impairments or impairments that were secondary to sensory problems, physical limitations, or environmental distractions were classified as cognitively unimpaired (CU). While we did not use thresholds, participants that demonstrated moderate impairments (e.g., −1.5 < *z* ≤ −1.0 based on UDS 3 norms^31^) on <3 neuropsychological tests and had no self- or other-reported of memory or thinking problems were classified as mildly impaired in cognition only (MI). The MI group reflects a group of individuals with minor cognitive impairments that are minimally impactful on day-to-day functioning, and that the cognitive impairments are of limited to no concern. Finally, the classification of cognitively impaired (CI) was given to participants with memory or thinking problems, and met one of the following conditions: (1) severe (e.g., *z* ≤ −1.5) impairment in at least one domain, plus at least moderate impairment in another cognitive domain, or (2) moderate impairment on more than 3 cognitive tests. When opinions of the panel varied, the data were reviewed and discussed until a consensus classification was reached.

### 2.3 | Cognitive tests

The cognitive battery used in our OOA study consists of standardized measures of memory, processing speed, language, and executive functioning. The tests were drawn from the Consortium to Establish a Registry for Alzheimer’s Disease neuropsychological assessment battery^32,33^ and supplemented by measures from the National Alzheimer’s Disease Coordinating Center (NACC) Uniform Data Set 2.^34^ These tests included Constructional Praxis copy (CP-copy) and Constructional Praxis recall (CP-recall)^35^; Logical Memory immediate (LM-I) and Logical Memory delay (LM-II)^34,36^; Modified Mini-Mental State Examination (3MS)^37^; Multilingual Naming Test (MINT)^38^; Trail Making Test part A (TMT-A) and Trail Making Test part B (TMT-B)^34,39^; Verbal Fluency-Animals (VF)^34^; Word List Memory Task immediate (WLM-immediate) and delay (WLM-delay).^32,33^ The cognitive measures are described in Supplementary Text S1: Cognitive Tests.

### 2.4 | Data processing

The cognitive measures above were used to develop the cognitive phenotype models. To optimize model development the following data processing steps were used to ensure data quality. First, outliers that may negatively affect the distribution of the data were winsorized, a process where one reduces the magnitude of the outliers by assigning them a value that is still at the high end of the distribution, but not as extreme.^40^ All cognitive tests were screened for outliers (>3 SD from the mean), which were then winsorized to 3 SD. This resulted in winsorization of 14 low scores on the 3MS, four low scores on the CP-Copy test, seven low scores on the MINT, 12 high scores on the TMT-A, five low scores on the WLM-immediate, two high scores on LM-I, and three high scores and one low score from VF. No scores were winsorized on the CP-Recall, LM-II, TMT-B, and WLM-Delay tests. Following winsorization, the cognitive tests were screened for non-normality and ceiling effects. Cognitive tests demonstrating non-normality (i.e., skewness and kurtosis< −2.5 or >2.5) or a ceiling effect (>25% of responses at range maximum) were excluded. The 3MS was excluded due to high kurtosis (2.71), and the CP-Copy test was excluded due to a ceiling effect (48.7% reached the maximum score).

Next, we addressed missing data (i.e., participants who did not complete all the cognitive tests). Of the 713 participants enrolled for the study, 22 participants completed zero of the cognitive tests, nine participants completed 1–3 tests, 24 participants completed 4–6 tests, 43 participants completed 7–8 tests, and 615 participants completed all nine of the included tests. For those with missing tests, we established a cutoff (participants with four or more tests [n = 67]) to ensure that there would be sufficient information to implement a predictive mean matching imputation via MICE R.^41^ This resulted in 682 participants (67 participants imputed data plus 615 participants with complete data) who were included in the analysis after imputation as follows: 25 (3.7%) data points from CP-Recall were imputed, 25 (3.7%) for LM-I, 25 (3.7%) for LM-II, 28 (4.1%) for MINT, 13 (1.9%) for TMT-A, 21 (3.1%) for TMT-B, six (0.9%) for WLM-delayed, zero for WLM-immediate, and 22 (3.2%) for VF.

Finally, age- and gender-related adjustments were completed when they were significant predictor variables. The adjustments were done using linear regression beta weights and intercepts, to create individual *Z*-scores for each of the cognitive tests.^42^ Due to the homogeneity in education (2.9% had less than 8 years, 93.5% had 8 years, and 3.5% had between 9 and 12 years), education was not adjusted. Age was a significant predictor on all tests and was adjusted across all tests. Gender was a significant predictor and adjusted for on the MINT, WLM-Delay, WLM- Immediate, and VF tests. If both age and gender were significant then both were entered into the model, otherwise only age was entered. See Supplementary Table S1 for adjustment beta weights.

### 2.5 | Construction of cognitive phenotypes

Four cognitive phenotype models were created including a global threshold model, global cluster model, memory threshold model, and memory cluster model. The global threshold model used the average *Z*-score of all cognitive tests, and participants were classified as *Above* (Avg *Z*-score > 0.5), *Average* (Avg *Z*-score ≤ 0.5; ≥−0.5), or *Below* (Avg *Z*-score < −0.5). The global cluster model is derived from a 4-means cluster analysis using the average *Z*-score. A 4-means cluster analysis was identified as having the best fit via the elbow method (number of clusters where the within-cluster sum of square is minimized and adding in additional clusters does not significantly improve the within-cluster sum of square) in the factoextra R package^43^ (Supplementary Figure S1). The four clusters were labeled *Far Below*, *Below*, *Average*, and *Above*. The memory threshold model used tests that assess the memory domain (memory: WLM-immediate, WLM-delay, CP-Recall, LM-I, LM-II;), and classified participants using the average memory *Z*-Score (*Above:* Avg *Z*-score > 0.5, *Average:* Avg *Z*-score ≤ 0.5; ≥ −0.5, *Below:* Avg *Z*-score < −0.5). Finally, the domain cluster model used the memory domain *Z*-score as the clustering variables. A 4-means cluster analysis was used after identifying four clusters as having the best fit using the elbow method^43^ (Supplementary Figure S2). The four clusters were labeled *Far Below*, *Below*, *Average*, and *Above*.

### 2.6 | Statistical analysis

To determine the best cognitive phenotype model, we examined the validity of the models and their ability to differentiate individuals based on their clinical classification and *APOE-e4* carrier status. An ordinal regression was run for each phenotype model (threshold models have 3 ordinal classification, and cluster models have 4 ordinal classification) with the independent variables of clinical classification (CI = 0, MI = 1, CU = 2), *APOE-e4* (present/absent), age, and sex (male/female). One-way ANOVAs for all models were used to examine cognitive phenotype differences on the proportion of CU, *APOE-e4*, age, sex. In addition, the models will have their phenotypes compared with one-way ANOVAs on four theory-driven cognitive domains (average *Z*-score for each of the following domains; memory: WLM-immediate, WLM-delay, CP-Recall, LM-I, LM-II; executive function: TMT-B; processing speed: TMT-A; and language: VF and MINT). Significance was set at *alpha* = 0.05. Tukey’s correction for multiple comparisons were used for one-way ANOVA post hoc tests.

## 3 | RESULTS

A total of 682 OOA individuals (mean age and SD 82.0 ± 4.1 years, 59.2% female) were included in the analysis. 24.6% were classified as CI, 13.9% were MI, and 61.5% were CU. *APOE-e4* data was available for 589 participants and 23.4% of those carried at least one *APOE-e4* allele. Only 5 (0.8%) individuals carried two APOE-e4 alleles and were not analyzed separately. Most of our participants (93.5%) had 8 years of education, 2.9% had less than 8 years, and 3.5% had between 9 and 12 years. Table 1 shows the demographic information and average performance on nine cognitive tests used in the analysis.

### 3.1 | Cognitive phenotype ordinal regression

The ordinal regression demonstrated that the memory cluster model showed the best fit (*χ*^*2*^ = 491.66, *R*^*2*^ = 0.55, *p* < 0.001). The memory threshold model had the next best fit (*χ2* = 394.35, *R*^*2*^ = 0.56, *p* < 0.001). The global cluster (*χ2* = 380.74, *R*^*2*^ = 0.53, *p* < 0.001) and global threshold (*χ2* = 301.11, *R*^*2*^ = 0.47, *p* < 0.001) models had poorer fits. Table 2 shows the number of participants, percent of each clinical classification (CI, MI or, CU), percent of *APOE-e4* carriers, sex, and age for each group in the memory cluster model (See Supplementary Table S2 for participant characteristics in each of the 4 models).

### 3.2 | Memory clusters

Four distinct cognitive groups were identified in the memory cluster analysis including a higher performing *Above* group, an average performing *Average* group, and two low performing groups (*Below* and *Far Below*). The clinical classification significantly contributed to the memory cluster model. Both CI (*log-odds* = −4.90, *Wald* = 238.57, *p* < 0.001) and MI (*log-odds* = −2.66, *Wald* = 100.58, *p* < 0.001) participants were more likely to be in worse performing clusters. The absence of the *APOE-e4* allele (*log-odds* = 0.61, *Wald* = 9.85, *p* = 0.002) and increase in age (*log-odds* = 0.11, *Wald* = 25.44, *p* < 0.001) were associated with being in a better performing cluster. Sex did not significantly contribute to the model (*log-odds* = 0.20, *Wald* = 1.49, *p* = 0.222). Tests of parallel lines led us to accept the assumption of proportional odds. The results of the ordinal regressions for all models are shown in Table 3.

**FIGURE 1:**
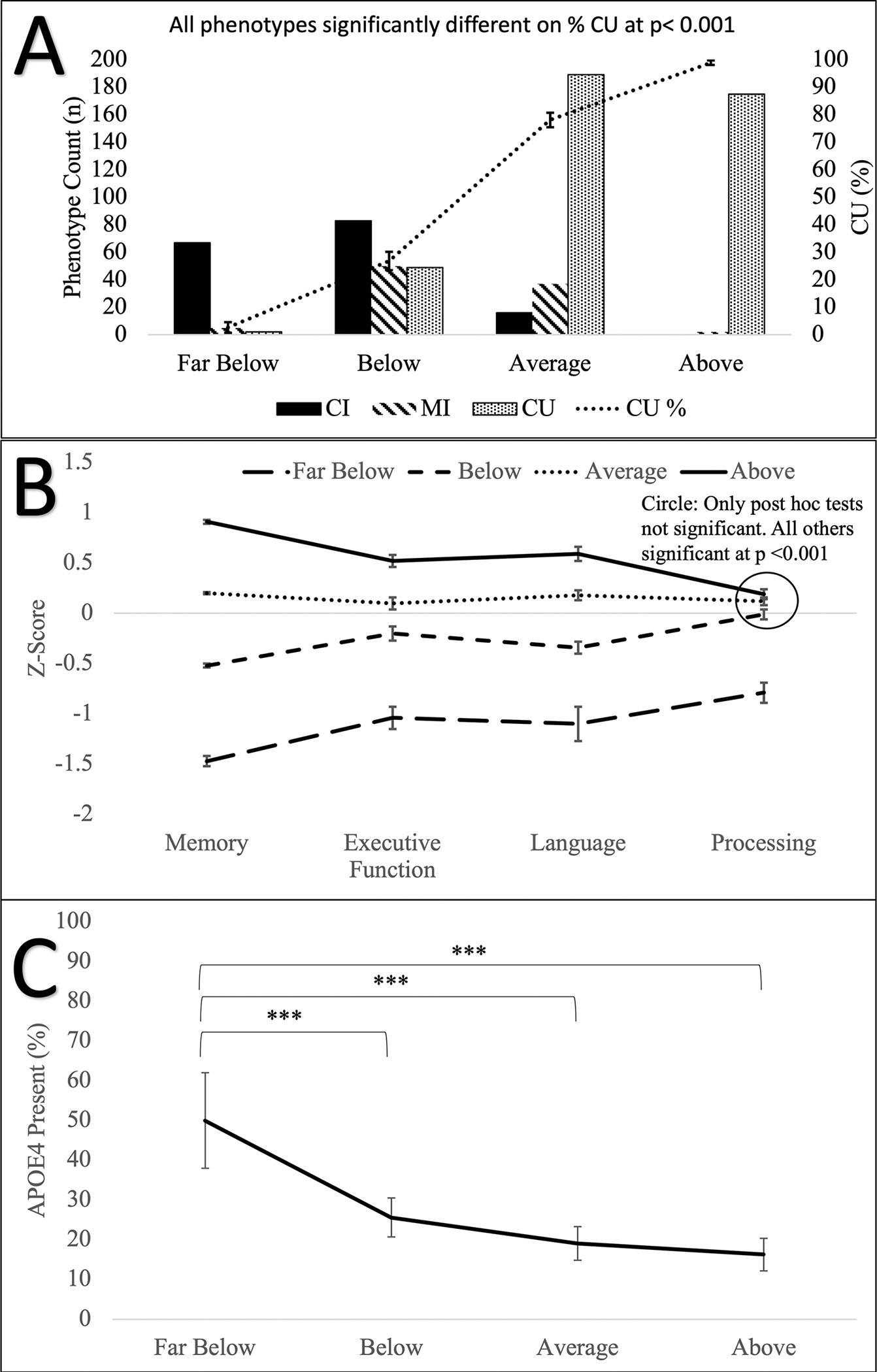
Memory cluster phenotypes one-way ANOVA results (A) Number of CI, MI, and CU individuals (left axis), and % CU (right axis). (B) *Z*-scores for each domain. (C) Percent of individuals with APOE-e4 allele. Standard error bars shown. CI, Cognitively Impaired; CU, Cognitively Unimpaired; MI, Mildly Impaired. *p < 0.05; **p < 0.01; ***p < 0.001.

A one-way ANOVA comparing the memory clusters demonstrated a main effect of phenotype on % CU (*F*_(*3, 674*)_ = 217.06, *p* < 0.001), *APOE-e4* (*F*_(*3,588*)_ = 11.33, *p* < 0.001), executive function (*F*_(*3,681*)_ = 46.93, *p* < 0.001), language (*F*_(*3,681*)_ = 140.06, *p* < 0.001), memory (*F*_(*3,681*)_ = 1740.26, *p* < 0.001), processing speed (*F*_(*3,681*)_ = 21.35, *p* < 0.001). All phenotypes were significantly different from each other at *p* < 0.001, with the exception of processing speed. Individuals in the *Far Below* phenotype demonstrated worse processing speed than the other three phenotypes (*p* < 0.001), and no significant differences were found between the other three phenotypes. No main effect of phenotype was demonstrated for age (*F*_*(3, 3681)*_ = 2.23, *p* = 0.083) or sex (*F*_*(3, 681)*_ = 0.28, *p* = 0.840). Figure 1 and Supplementary Table S3 show the one-way ANOVA results. Supplementary Tables S4–S6 show the one-way ANOVA results for the other models.

## 4 | DISCUSSION

Using psychometric methods, we created and compared four models to derive cognitive phenotypes. The model with the best fit was the memory clustering model (*R*^2^ = 0.55). As reported above, this method yielded four phenotypes labeled as *Above*, *Average*, *Below*, and *Far Below*, each of which aligned with clinical classifications. The *Above* phenotype had the greatest percentage of CU individuals, while the *Below* and *Far Below* phenotypes consisted predominantly of CI individuals. We also found a linear relationship between the cognitive phenotypes and APOE-e4 prevalence, which was significantly higher in the *Far Below* phenotype. These results suggest that our phenotypes are in agreement with the clinical classifications and *APOE-e4* genotypes. Finally, in our logistic regression we found that older individuals were more likely to be stratified into the better performing phenotypes, however the effect size (*log-odds* = 0.11) was small, and a one-way ANOVA showed that there were no age differences. Thus, this approach both aligned with clinical classifications and more finely differentiated CU individuals into two separate phenotypes (*Above* and *Average*) which will be useful for subsequent genetic analyses. Importantly, compared to the individuals in the *Average* phenotype, individuals in the *Above* phenotype performed better on tests of executive function, memory, and language. We expect that individuals in the *Above* phenotype have genetic variants that preclude them from experiencing substantial declines in multiple domains of cognition.

Overall, the memory models had a better fitthan the global models. Domain based stratification is commonly used in detecting genetic variants associated with AD,^4,5^ and in this study memory stratifications aligned with clinical adjudications the best. However, the global stratifications also demonstrated good fit with the clinical adjudications. Factor analyses find about 40% of the variance of subjects’ scores on any given cognitive test can be explained by a global factor,^22^ and hundreds of independent loci have been associated with global cognition.^44^ Both methods of stratification may be useful in searching for genetic variants depending on the phenotypes of interest.

In addition to the results above, we found that several individuals were discordant for clinical classification and phenotype (See Supplementary Table S7). For instance, two MI individuals were stratified into our *Above* phenotype, two CU individual stratified in the *Far Below* phenotype, and 49 (11.8%) CU individuals stratified in the *Below* phenotype. This suggests that cognitive performance alone offers a different perspective versus when cognitive performance is merged with other information in the clinical classification process. The discrepancies are most likely due to other influences on clinical classification decision making (e.g., consideration of sensory or motor problems, weighing functional disabilities, non-memory cognitive impairment, and subjective memory complaints). Given the risk for cognitive problems associated with age, the test performances of MI individuals in the *Above* phenotype suggests that they are experiencing protective benefits in memory. Further, in the context of the larger project, individuals in the *Above* phenotype may be more likely to have genetic variants that protect them from significant memory decline.

Finally, the phenotypes identified in this analysis are relative to one another based on *Z*-scores derived from the OOA. However, when compared to age-, sex, and education-adjusted population norms using the NACC normative calculator,^42^ our *Above* phenotype had lower *Z*-scores relative to the normal population (i.e., our *Above* phenotype fell in the average range across several measures). Thus, identifying high performers using normative data in the OOA may be less accurate. This is not surprising given the unique educational, occupational, and lifestyle factors in the OOA.

### 4.1 | Limitations

For this study we identified four cognitive phenotypes in our OOA participants. As noted above, ~7.8% of participants displayed a discordance between cognitive phenotype and clinical classifications. Along these lines, it would be useful to look at physical and sensory comorbidities in our sample. Specifically, did participants have increased visual, hearing, or motor difficulties that, while not substantial, could have affected test performance. For example, visual, hearing, and motor (e.g., osteoarthritis of the hand) impairments are common in older adults and could impact their test performance.^45–47^ While the phenotypes created in this study were based on memory and global cognition, there may be additional value in exploring phenotypes based on other cognitive domains. For example, preliminary data in our OOA population suggest that there is considerable sparing of visual construction and visuospatial memory skills. Performance on this test may be informative with respective to genetic or lifestyle factors resulting in preservation of visuospatial abilities. Another consideration is that the models could be affected by the test battery, and global threshold and clusters models might show a better fit with larger test batteries with more comprehensive testing in non-memory domains or a larger sample size. Similarly, the homogeneity of our OOA sample may limit the generalizability of this psychometric approach to defining cognitive phenotypes in the general population as well as other unique populations.

### 4.2 | Conclusion

We found that the memory cluster model that produced four distinct phenotypes had the best fit with clinical classification, presence of *APOE-e4*, age, and sex in our OOA sample. This suggests that stratification of OOA individuals using clustering of memory performs better than threshold stratification, and stratification based on global cognition. However, we found that several individuals were discordant for their clinical classification and phenotypes. The discrepancies found are likely related to non-cognitive information that is a part of the clinical classification process (e.g., memory complaints and functional disabilities). Importantly, our memory cluster model stratified CU individuals into two distinct phenotypes with significant differences in cognitive performance in three of the domains (memory, executive functioning, and language), but similar prevalence frequencies of *APOE-e4.* Overall, this suggests that individuals in the *Above* phenotype may have genetic variants that protect cognition and memory.

## Supplementary Material

Supplementary Materials

## Figures and Tables

**TABLE 1:** Means and standard deviations of demographic and cognitive variables.

Demographic variables		Cognitive variables (*n* = 682)	

Age (*n* = 682)	82.0 ± 4.1 years	VF	15.7 ± 4.9
Gender (*n* = 682)	59.2% female	WLM-delay	4.8 ± 2.5
Education (*n* = 682)	93.5% with 8 years	WLM-immediate	16.8 ± 4.6
*APOE-e4* (*n* = 589)	23.4% with *APOE-e4*	CP-recall	7.7 ± 2.9
Clinical classification (*n* = 675)	61.5% CU 13.9% MI 24.6% CI	TMT-A	59.1 ± 29.3
		TMT-B	173.5 ± 73.3
		MINT	27.3 ± 2.7
		LM-I	8.8 ± 4.3
		LM-II	8.2 ± 4.4

*Note*: Standard deviations shown.

Abbreviations: CI, Cognitively Impaired; CP, Constructional Praxis; CU, Cognitively Unimpaired; LM, Logical Memory; MI, Mildly Impaired; MINT, Multi-Lingual Naming Test; TMT, Trail Making Test; WLM, Word List Memory; VF, Verbal Fluency.

**TABLE 2:** Participant characteristics in the memory cluster phenotypes.

Model	Group	CU (%)	MI (%)	CI (%)	*APOE-e4* (%)	Female (%)	Age (SE)

Memory clusters	*Far below* (*n* = 77)	2.7	6.8	90.5	50.0	58.4	82.7 (0.4)
	*Below* (*n* = 185)	26.9	27.5	45.6	25.6	58.4	82.0 (0.3)
	*Average* (*n* = 243)	78.1	15.3	6.6	19.1	62.2	81.5 (0.2)
	*Above* (*n* = 177)	98.9	1.1	0.0	16.3	59.2	82.3 (0.3)

Abbreviations: CI, Cognitively Impaired; CU, Cognitively Unimpaired; MI, Mildly Impaired; SE, Standard error.

**TABLE 3:** Ordinal regression comparing cognitive phenotype models (*n* = 582).

Model	Global threshold	Global cluster	Memory threshold	Memory cluster

Classification (CI)	−4.37, 173.41	−4.64, 224.31	−4.97, 189.85	−4.90, 238.57
	*p* < 0.001	*p* < 0.001	*p* < 0.001	*p* < 0.001
Classification (MI)	−2.10, 43.22	−2.68, 98.69	−3.44, 97.64	−2.66, 100.58
	*p* < 0.001	*p* < 0.001	*p* < 0.001	*p* < 0.001
*APOE-e4* (Absent)	0.68, 9.60	0.61, 9.263	0.71, 11.20	0.61, 0.85
	*p* = 0.002	*p* = 0.002	*p* < 0.001	*p* = 0.002
Age	0.10, 18.58	0.11, 24.58	0.12, 27.27	0.11, 25.44
	*p* < 0.001	*p* < 0.001	*p* < 0.001	*p* < 0.001
Gender (Male)	0.00, 0.00	0.21, 1.59	0.14, 0.60	0.20, 1.49
	*p* = 0.995	*p* = 0.207	*p* = 0.439	*p* = 0.222
Model (χ^2^)	301.11	380.74	394.35	419.66
	*p* < 0.001	*p* < 0.001	*p* < 0.001	*p* < 0.001
Pseudo *R*^2^ (Nagelkerke)	*R*^2^ = 0.47	*R*^2^ = 0.53	*R*^2^ = 0.56	*R*^2^ = 0.55

*Note:* Statistics shown are Log-Odds, Wald, and *p* unless otherwise noted.

Abbreviations: CI, Cognitively Impaired; MI, Mildly Impaired.

## Data Availability

The data that support the findings of this study are available from the corresponding author upon reasonable request.

## Key points

- This study compares psychometric methods for developing cognitive phenotypes that are more granular than clinical classifications in individuals from the Old Order Amish.
- We found that the memory cluster model produced four distinct cognitive phenotypes had the best fit with clinical classification, presence of *APOE-e4*, age, and sex in the Old Order Amish.
- This suggests that stratification of Old Order Amish individuals using cluster analysis based on memory performance performs better than memory threshold stratification, and stratification based on global cognition.
- Importantly, our memory cluster model stratified individuals into four distinct cognitive phenotypes with significant differences in cognitive performance across memory, executive functioning, and language, but similar prevalence rates of *APOE-e4* in top three performing phenotypes
