# Supplementary material for: Psychometric approaches to defining cognitive phenotypes in the Old Order Amish
Source: Int J Geriatr Psychiatry. Author manuscript; Available in PMC 2025 Apr 16. (PMC12000893; doi:10.1002/gps.5903)
Supplement: Supplementary Materials [file NIHMS2068536-supplement-Supplementary_Materials.docx]

**Supplemental Materials**

Supplementary Text: Cognitive Tests

*The Modified Mini-Mental State Examination (3MS)*

The 3MS [1] is a widely used measure dementia screening measure that assesses multiple cognitive abilities (attention, executive function, language, memory, orientation, and visuospatial). The 3MS yields a total score that ranges from 0-100.

*Constructional Praxis (CP)*

The CP test measures immediate and delayed visuospatial and constructional abilities [2]. For this test, participants copy four-line drawings (i.e., circle, diamond, overlapping rectangles, and cube) and, following a delay, are then asked to reconstruct the four-line drawings from memory. Both the CP-copy and CP-recall tasks are scored using standard criteria and scores range from 0-11.

*Logical Memory Test (LM)*

The Logical Memory test consists of an immediate recall and delayed recall tasks [3,4]. Participants are read a short three sentence story and asked to recall the story immediately (LM-I) and after a 20-minute delay (LM-II). Their recall is scored using standard criteria and scores range from 0-25.

*Multilingual Naming Test (MINT)*

The Multilingual Naming Test (MINT) is a naming test that was developed for use in four different languages (i.e., English, Spanish, Mandarin, Hebrew) with equivalent overall difficulty across languages [5,6]. During the abbreviated MINT, participants are asked to name 32-line drawings of objects presented one at a time [7]. If the person is unable to name the object, a semantic cue is provided. If the individual is still unable to name the object, a phonemic cue is provided. The total score (0-32) is the sum of correct spontaneous responses and semantic cue responses.

*Trail Making Test (TMT)*

TMT parts A and B are used as tests of processing speed and executive functioning respectively [3,8]. When performing the TMT-A, participants draw a line from one number to the next in ascending order (starting at 1 and ending at 25) for a total of 24 lines. In the TMT-B, participants draw a line between alternating numbers and letters in ascending order (1-A-2-B, etc.) until they reached the number 13 for a total of 24 lines. Participants are given a maximum of 300 seconds to complete the task; for those who take longer, scores are truncated to 300 seconds.

*Word List Memory Task (WLM)*

The WLM is a 10-item list learning task that has both immediate and delayed recall tasks [9,10]. For the immediate recall task, participants are presented with 10 printed words and asked to read each word out loud as it was presented. One word is presented every two seconds. After the 10 words are presented, the participant is given 90 seconds to recall as many words as possible. There are three learning trials, and the same 10 words are presented each time in a different order. The WLM-immediate score is the sum of the three trials for a maximum score of 30. The WLM-delayed score quantifies the ability of the participant to recall the 10 words after a delay of 15 minutes. The maximum delayed score is 10.

*Verbal Fluency - Animal (VF)*

The VF is a measure of language [3]. During the VF animal naming task participants are asked to name as many different animals as possible (real or imaginary) in 60 seconds. No points are given for proper nouns or repetitions.

Tables

| Supplementary Table 1: Regression Beta Weights For Age and Sex Adjustments | | |
| --- | --- | --- |
|  | Age (years) | Sex (Female) |
| CP-Recall | *B* = -0.170, *p* < 0.001 | *NS* |
| LM-I | *B* = -0.272, *p* < 0.001 | *NS* |
| LM-II | *B* = -0.299, *p* < 0.001 | *NS* |
| Mint | *B* = -0.177, *p* < 0.001 | *B* = -1.144, *p* < 0.001 |
| TMT-A | *B* = 2.755, *p* < 0.001 | *NS* |
| TMT-B | *B* = 7.480, *p* < 0.001 | *NS* |
| WLM-delayed | *B* = -0.160, *p* < 0.001 | *B* = 0.694, *p* < 0.001 |
| WLM-immediate | *B* = -0.297, *p* < 0.001 | *B* = 1.471, *p* < 0.001 |
| VF | *B* = -0.387, *p* < 0.001 | *B* = -0.770, *p* = 0.03 |
| Note: *B*: Unstandardized beta; CP: Constructional Praxis; LM: Logical Memory; MI: Mildly Impaired; MINT: Multi-Lingual Naming Test; TMT: Trail Making Test; WLM: Word List Memory; *NS*: Not Significant; VF: Verbal Fluency | | |

| Supplementary Table 2: Participant characteristics in each of the 4 models | | | | | | | |
| --- | --- | --- | --- | --- | --- | --- | --- |
| Model | Group | \| CU (%) \| % Borderline \| % Impaired \| % APOE4 \| % Female \| Age \| \| --- \| --- \| --- \| --- \| --- \| --- \| | MI (%) | CI (%) | *APOE-e4* (%) | Female  (%) | Age  (SE) |
| Global Threshold | *Below*  (n = 138) | 7.5 | 13.4 | 79.1 | 40.7 | 58.7 | 82.9 (0.4) |
|  | *Average*  (n= 385) | 66.5 | 17.8 | 15.7 | 21.3 | 57.1 | 81.3 (0.2) |
|  | *Above*  (n = 159) | 95.0 | 5.0 | 0.0 | 14.7 | 64.8 | 82.7 (0.3) |
| Global Clusters | *Far Below*  (n = 39) | 2.7 | 2.7 | 94.6 | 50.0 | 69.2 | 83.1 (0.7) |
|  | *Below*  (n = 190) | 16.1 | 26.3 | 57.5 | 31.5 | 54.7 | 82.1 (0.3) |
|  | *Average*  (n = 256) | 76.9 | 14.1 | 9.0 | 19.8 | 59.0 | 81.5 (0.2) |
|  | *Above*  (n = 197) | 95.4 | 4.1 | 0.5 | 15.6 | 61.9 | 82.2 (0.3) |
| Memory Threshold | *Below*  (n = 170) | 7.9 | 20.7 | 71.3 | 39.9 | 56.5 | 82.5 (0.3) |
|  | *Average*  (n= 313) | 66.4 | 18.0 | 15.7 | 19.7 | 60.1 | 81.5 (0.2) |
|  | *Above*  (n = 199) | 98.0 | 2.0 | 0.0 | 15.9 | 60.3 | 82.3 (0.3) |
| Memory Clusters | *Far Below*  (n = 77) | 2.7 | 6.8 | 90.5 | 50.0 | 58.4 | 82.7 (0.4) |
|  | *Below*  (n = 185) | 26.9 | 27.5 | 45.6 | 25.6 | 58.4 | 82.0 (0.3) |
|  | *Average*  (n = 243) | 78.1 | 15.3 | 6.6 | 19.1 | 62.2 | 81.5 (0.2) |
|  | *Above*  (n = 177) | 98.9 | 1.1 | 0.0 | 16.3 | 59.2 | 82.3 (0.3) |
| *Note.* CU: Cognitively Unimpaired; CI: Cognitively Impaired; MI: Mildly Impaired; SE: Standard error. | | | | | | | |

| Supplementary Table 3: Memory Clusters One-Way ANOVA | | | | |
| --- | --- | --- | --- | --- |
|  | *Above* | *Average* | *Below* | *Far Below* |
| CU (%) | 98.9 ± 0.8  ^aaa,bbb,ccc^ | 78.1 ± 2.7  ^ccc,ddd,eee^ | 26.9 ± 3.3  ^bbb,eee,fff^ | 2.7 ± 1.9  ^aaa,ddd,fff^ |
| *APOE-e4* (%) | 16.3 ± 2.9  ^aaa^ | 19.1 ± 2.7  ^ddd^ | 25.6 ± 3.5  ^fff^ | 50.0 ± 6.3  ^aaa,ddd,fff^ |
| Age | 82.28 ± 0.30 | 81.49 ± 0.25 | 82.04 ± 0.32 | 82.65 ± 0.45 |
| Female (%) | 62.2 ± 3.7 | 58.0 ± 3.2 | 58.4 ± 3.6 | 58.4 ± 5.7 |
| Processing Speed  (Z-Score) | 0.19 ± 0.07  ^aaa^ | 0.12 ± 0.05  ^ddd^ | -0.01 ± 0.06  ^fff^ | -0.79 ± 0.17  ^aaa,ddd,fff^ |
| Executive Function  (Z-Score) | 0.52 ± 0.06  ^aaa,bbb,ccc^ | 0.10 ± 0.06  ^ccc,ddd,eee^ | -0.20 ± 0.07  ^bbb,eee,fff^ | -1.04 ± 0.11  ^aaa,ddd,fff^ |
| Language  (Z-Score) | 0.59 ± 0.05  ^aaa,bbb,ccc^ | 0.18 ± 0.04  ^ccc,ddd,ee^ | -0.34 ± 0.05  ^bbb,ee,fff^ | -1.10 ± 0.10  ^aaa,ddd,fff^ |
| Memory  (Z-Score) | 0.91 ± 0.02  ^aaa,bbb,ccc^ | 0.20 ± 0.01  ^ccc,ddd,eee^ | -0.52 ± 0.02  ^bbb,eee,fff^ | -1.47 ± 0.05  ^aaa,ddd,fff^ |
| *Note.* Means and standard errors shown.  Difference between *Above Average* and *Far Below Average*: ^aaa^ = *p* < 0.001  Difference between *Above Average* and *Below Average*: ^bbb^ = *p* < 0.001  Difference between *Above Average* and *Average*: ^ccc^ = *p* < 0.001  Difference between *Average* and *Far Below Average*: ^ddd^ = *p* < 0.001  Difference between *Average* and *Below Average*: ^ee^ = *p* <0.01, ^eee^ = *p* < 0.001  Difference between *Below Average* and *Far Below Average*: ^fff^ = *p* < 0.001 | | | | |

| Supplementary Table 4: Global Clusters One-Way ANOVA | | | | |
| --- | --- | --- | --- | --- |
|  | *Above* | *Average* | *Below* | *Far Below* |
| CU (%) | 95.4 ± 1.5  ^aaa,bbb,ccc^ | 76.9 ± 2.6  ^ccc,ddd,eee^ | 16.1 ± 2.7  ^bbb,eee^ | 2.7 ± 2.7  ^aaa,ddd^ |
| *APOE-e4* (%) | 15.6 ± 2.7  ^aaa,bb^ | 19.8% ± 2.7  ^ddd,e^ | 31.5 ± 3.6  ^bb,e^ | 50.0 ± 9.0  ^aaa,ddd^ |
| Age | 82.24 ± 0.29 | 81.48 ± 0.25 | 82.14 ± 0.31 | 83.05 ± 0.65 |
| Female (%) | 61.9 ± 3.5 | 59.0 ± 3.1 | 54.7 ± 3.6 | 69.2 ± 7.5 |
| Processing Speed  (Z-Score) | 0.48 ± 0.04  ^aaa,bbb,ccc^ | 0.09 ± 0.05  ^ccc,ddd,eee^ | -0.25 ± 0.07  ^bbb,eee,fff^ | -1.77 ± 0.25  ^aaa,ddd,fff^ |
| Executive Function  (Z-Score) | 0.73 ± 0.05  ^aaa,bbb,ccc^ | 0.09 ± 0.05  ^ccc,ddd,eee^ | -0.54 ± 0.06  ^bbb, eee, fff^ | -1.66 ± 0.07  ^aaa,ddd,fff^ |
| Language  (Z-Score) | 0.71 ± 0.04  ^aaa,bbb,ccc^ | 0.10 ± 0.03  ^ccc, ddd, eee^ | -0.52 ± 0.04  ^bbb, eee, fff^ | -1.74 ± 0.12  ^aaa,ddd,fff^ |
| Memory  (Z-Score) | 0.80 ± 0.03  ^aaa,bbb,ccc^ | 0.12 ± 0.02  ^ccc ddd,eee^ | -0.65 ± 0.03  ^bbb,eee,fff^ | -1.67 ± 0.07  ^aaa,ddd,fff^ |
| *Note.* Means and standard errors shown.  Difference between *Above Average* and *Far Below Average*: ^aaa^ = *p* < 0.001  Difference between *Above Average* and *Below Average*: ^bb^ = *p* < 0.01^bbb^ = *p* < 0.001  Difference between *Above Average* and *Average*: ^ccc^ = *p* < 0.001  Difference between *Average* and *Far Below Average*: ^ddd^ = *p* < 0.001  Difference between *Average* and *Below Average*: ^e^ = *p* < 0.05, ^eee^ = *p* < 0.001  Difference between *Below Average* and *Far Below Average*: ^fff^ = *p* < 0.001 | | | | |

| Supplementary Table 5: Memory Threshold One-Way ANOVA | | | |
| --- | --- | --- | --- |
|  | *Above* | *Average* | *Below* |
| CU (%) | 98.0 ± 1.0  ^bbb,ccc^ | 66.4 ± 2.7  ^ccc,eee^ | 7.9 ± 2.1  ^bbb,eee^ |
| *APOE-e4* (%) | 15.9 ± 2.7  ^bbb^ | 19.7 ± 2.5  ^eee^ | 39.9 ± 4.1  ^bbb,eee^ |
| Age | 82.31 ± 0.29 | 81.49 ± 0.23  ^e^ | 82.46 ± 0.32  ^e^ |
| Female (%) | 60.3 ± 3.5 | 60.1 ± 2.8 | 56.5 ± 3.8 |
| Processing Speed  (Z-Score) | 0.16 ± 0.06  ^bbb^ | 0.11 ± 0.05  ^eee^ | -0.38 ± 0.10  ^bbb,eee^ |
| Executive Function  (Z-Score) | 0.46 ± 0.06  ^bbb,ccc^ | 0.04 ± 0.05  ^ccc,eee^ | -0.61 ± 0.08  ^bbb,eee^ |
| Language  (Z-Score) | 0.54 ± 0.04  ^bbb,ccc^ | 0.06 ± 0.04  ^ccc,eee^ | -0.74 ± 0.06  ^bbb,eee^ |
| Memory  (Z-Score) | 0.86 ± 0.03  ^bbb,ccc^ | 0.03 ± 0.02  ^ccc,eee^ | -1.06 ± 0.04  ^bbb,eee^ |
| *Note.* Means and standard errors shown.  Difference between *Above Average* and *Below Average*: ^bbb^ = *p* < 0.001  Difference between *Above Average* and *Average*: ^ccc^ = *p* < 0.001  Difference between *Average* and *Below Average*: ^e^ = *p* < 0.05, ^eee^ = *p* < 0.001 | | | |

| Supplementary Table 6: Global Threshold One-Way ANOVA | | | |
| --- | --- | --- | --- |
|  | *Above* | *Average* | *Below* |
| CU (%) | 95.0 ± 1.7  ^bbb,ccc^ | 66.5 ± 2.4  ^ccc,eee^ | 7.5 ± 2.3  ^bbb,eee^ |
| *APOE-e4* (%) | 14.7 ± 3.0  ^bbb^ | 21.3 ± 2.2  ^eee^ | 40.7 ± 4.6  ^bbb,eee^ |
| Age | 82.68 ± 0.32  ^cc^ | 81.34 ± 0.20  ^cc,eee^ | 82.94 ± 0.35  ^eee^ |
| Female (%) | 64.8 ± 3.8 | 57.1 ± 2.5 | 58.7 ± 4.2 |
| Processing Speed  (Z-Score) | 0.55 ± 0.05  ^bbb,ccc^ | 0.02 ± 0.04  ^ccc,eee^ | -0.68 ± 0.11  ^ccc,eee^ |
| Executive Function  (Z-Score) | 0.78 ± 0.06  ^bbb,ccc^ | 0.02 ± 0.04  ^ccc,eee^ | -0.96 ± 0.08  ^ccc,eee^ |
| Language  (Z-Score) | 0.78 ± 0.04  ^bbb,ccc^ | 0.04 ± 0.03  ^ccc,eee^ | -1.00 ± 0.07  ^ccc,eee^ |
| Memory  (Z-Score) | 0.87 ± 0.03  ^bbb,ccc^ | 0.04 ± 0.02  ^ccc,eee^ | -1.12 ± 0.04  ^ccc,eee^ |
| *Note.* Means and standard errors shown.  Difference between *Above Average* and *Below Average*: ^bbb^ = *p* < 0.001  Difference between *Above Average* and *Average*: ^cc^ = *p* < 0.01, ^ccc^ = *p* < 0.001  Difference between *Average* and *Below Average*: ^eee^ = *p* < 0.001 | | | |

| Supplementary Table 7: Adjudication and Memory Cluster Discordances | | | | |
| --- | --- | --- | --- | --- |
|  | Sample Average  (n = 682) | MI in *Above*  (n =2) | CU in  *Far Below* (n=2) | CU in *Below*  (n = 49) |
| Age | 82.0 | 86 | 80.5 | 79.67 |
| CP-Recall  (Z-Score) | 7.7 | 11.0 (1.41) | 4.5 (-1.19) | 6.6 (-0.52) |
| LM-I  (Z-Score) | 8.8 | 14.0 (1.54) | 4 (-1.27) | 7.8 (-0.40) |
| LM-II  (Z-Score) | 8.2 | 13.0 (1.42) | 5 (-0.85) | 7.5 (-0.33) |
| MINT  (Z-Score) | 27.3 | 28.0 (0.52) | 26.5 (-0.47) | 27.4 (-0.10) |
| TMT-A  (Z-Score) | 59.1 | 78.0 (-0.29) | 99 (-1.63) | 51.1 (0.06) |
| TMT-B  (Z-Score) | 173.5 | 159.0 (0.67) | 300 (-2.07) | 152.2 (0.06) |
| WLM-Delay  (Z-Score) | 4.8 | 6.0 (0.79) | 1 (-1.69) | 4.5 (- 0.32) |
| WLM-Immediate  (Z-Score) | 16.8 | 11.5 (-0.92) | 11 (-1.42) | 16.3 (-0.32) |
| VF  (Z-Score) | 15.7 | 16.0 (0.38) | 11.5 (-1.06) | 16.5 (-0.02) |
| *Note*. CI: Cognitively Unimpaired; CI: Cognitively Impaired; CP: Constructional Praxis; LM: Logical Memory; MI: Mildly Impaired; MINT: Multi-Lingual Naming Test; TMT: Trail Making Test; WLM: Word List Memory; VF: Verbal Fluency | | | | |

**Figures**

Supplementary Figure 1: The Elbow Method of k-means for the global Z-Scores. Line indicates inflection point, and 4 global clusters were identified.

Supplementary Figure 2: The Elbow Method of k-means for the memory Z-Scores. Line indicates inflection point, and four memory clusters were identified.

**References**

[1] Teng EL, Chui HC (1987) The Modified Mini-Mental State (3MS) examination. *J Clin Psychiatry* **48**, 314-318.

[2] Fillenbaum GG, Burchett BM, Unverzagt FW, Rexroth DF, Welsh-Bohmer K (2011) Norms for CERAD constructional praxis recall. *Clin Neuropsychol* **25**, 1345-1358.

[3] Weintraub S, Salmon D, Mercaldo N, Ferris S, Graff-Radford NR, Chui H, Cummings J, DeCarli C, Foster NL, Galasko D, Peskind E, Dietrich W, Beekly DL, Kukull WA, Morris JC (2009) The Alzheimer's Disease Centers' Uniform Data Set (UDS): the neuropsychologic test battery. *Alzheimer Dis Assoc Disord* **23**, 91-101.

[4] Wechsler D (1987) *Wechsler Memory Scale-Revised*, Psychological Corporation, San Antonio, Texas.

[5] Gollan TH, Weissberger GH, Runnqvist E, Montoya RI, Cera CM (2012) Self-ratings of Spoken Language Dominance: A Multi-Lingual Naming Test (MINT) and Preliminary Norms for Young and Aging Spanish-English Bilinguals. *Biling (Camb Engl)* **15**, 594-615.

[6] Stasenko A, Jacobs DM, Salmon DP, Gollan TH (2019) The Multilingual Naming Test (MINT) as a Measure of Picture Naming Ability in Alzheimer's Disease. *J Int Neuropsychol Soc* **25**, 821-833.

[7] Ivanova I, Salmon DP, Gollan TH (2013) The multilingual naming test in Alzheimer's disease: clues to the origin of naming impairments. *J Int Neuropsychol Soc* **19**, 272-283.

[8] Reitan RM, Wolfson D (1985) *THe Halstead-Reitan Neuropsychological Test Battery*, Neuropsychology Press, Tuscon, Arizona.

[9] Morris JC, Heyman A, Mohs RC, Hughes JP, Van Belle G, Fillenbaum G, Mellits ED, Clark C (1989) The Consortium to Establish a Registry for Alzheimer's Disease (CERAD): Part I. Clinical and neuropsychological assessment of Alzheimer's disease. *Neurology* **39**, 1159-1165.

[10] Fillenbaum GG, van Belle G, Morris JC, Mohs RC, Mirra SS, Davis PC, Tariot PN, Silverman JM, Clark CM, Welsh-Bohmer KA, Heyman A (2008) Consortium to Establish a Registry for Alzheimer's Disease (CERAD): the first twenty years. *Alzheimers Dement* **4**, 96-109.
